# Proteomic analysis of the ventral disc of *Giardia lamblia*

**DOI:** 10.1186/1756-0500-5-41

**Published:** 2012-01-19

**Authors:** Daniela Lourenço, Iamara da Silva Andrade, Letícia Labati Terra, Patricia Ramos Guimarães, Russolina Benedeta Zingali, Wanderley de Souza

**Affiliations:** 1 Instituto Nacional de Metrologia, Normalização e Qualidade Industrial - INMETRO, Rio de Janeiro, Brazil; 2 Laboratório de Ultraestrutura Celular Hertha Meyer, IBCCF, Universidade Federal do Rio de Janeiro, Rio de Janeiro, Brazil; 3 Unidade de Espectrometria de Massas e Proteômica, Instituto de Bioquímica Médica e Rede Proteômica do Rio de Janeiro, Universidade Federal do Rio de Janeiro, 21941-902 Rio de Janeiro, Brazil; 4 Instituto Nacional de Ciência e Tecnologia em Biologia Estrutural e Bioimagem, Rio de Janeiro, Brazil

## Abstract

**Background:**

*Giardia lamblia *is a multiflagellated protozoan that inhabits the small intestine of vertebrates, causing giardiasis. To colonize the small intestine, the trophozoites form of the parasite remains attached to intestinal epithelial cells by means of cytoskeletal elements that form a structure known as the ventral disc. Previous studies have shown that the ventral disc is made of tubulin and giardins.

**Results:**

To obtain further information on the composition of the ventral disc, we developed a new protocol and evaluated the purity of the isolation by transmission electron microscopy. Using 1D- and 2D-PAGE and mass spectrometry, we identified proteins with functions associated with the disc. In addition to finding tubulin and giardin, proteins known to be associated with the ventral disc, we also identified proteins annotated in the *Giardia *genome, but whose function was previously unknown.

**Conclusions:**

The isolation of the ventral disc shown in this work, compared to previously published protocols, proved to be more efficient. Proteomic analysis showed the presence of several proteins whose further characterization may help in the elucidation of the mechanisms involved in the attachment of the protozoan to epithelial cells.

## Background

*Giardia lamblia *is a multiflagellated protozoan parasite that colonizes the upper portion of the small intestine in humans and animals causing watery diarrhea, epigastic pain, nausea, vomiting and weight loss. It is one of the most prevalent intestinal parasites of humans and animals, and there are widespread reports of diarrhea caused by this protozoan. In developing countries, the prevalence of giardiasis is estimated to be 20-30% [1].

The life cycle of *G. lamblia *comprises two developmental stages: the infectious and environmentally-resistant cyst and a motile active trophozoite [2,3]. The cytoskeleton of *Giardia *is tightly connected to both differentiation and virulence; it is constantly required for parasite attachment, detachment, and movement in response to the changing conditions present in the intestine [4,5]. *G. lamblia *trophozoites display a remarkable, complex cytoskeleton that contains unique structures; it is perhaps the most striking feature of this parasite. The most outstanding constituents of this parasite cytoskeleton include four pairs of flagella, the funis, the median body and the ventral disc [5,6].

The ventral disc is unique to the *Giardia *parasite, it is not even present in the most closely related organisms of the order Diplomonadida [4]; therefore, the ventral disc may constitute a promising target for chemotherapy. The ventral disc covers the anterior half of the ventral side of the parasite, and it is a large, rigid structure composed of three distinct structures. First, there are microtubules, which are arranged in a coiled array around a bare area. Second, there are microribbons that protrude into the cytoplasm, and third, there are cross bridges that connect adjacent microtubules [7-10]. The ventral disc plays a fundamental role in parasite adhesion to intestinal epithelial cells. During the adhesion process, the ventral pair of flagella beat continuously in a sinusoidal wave motion that draws fluid out from under the ventral disc. This movement creates an area of low pressure, causing parasite adhesion to the intestinal epithelium [7,11]. In addition, the disc also plays some role in the process of nuclear division [12].

Several proteins have been identified in the *Giardia *cytoskeleton, most predominantly tubulin and giardins. Giardins are comprised of α-giardins, a member of a group of annexin-like proteins found only in *Giardia *[13], as well as β, γ and δ giardins. [3,5,14]. Another important protein, SALP-1 (striated fiber-assemblin-like protein), was identified and localized to the ventral disc of *Giardia *[15].

Here, we obtained an enriched fraction of isolated ventral discs and performed proteomic analyses using 1D and 2D-PAGE and mass spectrometry to further investigate its protein composition.

## Results

### Isolation of the ventral disc

**Figure 1:**
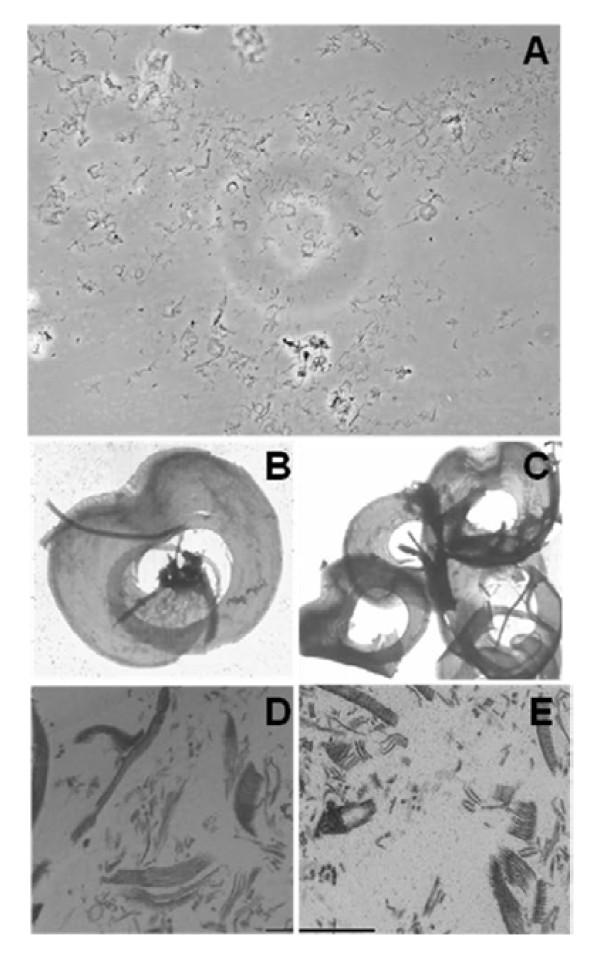
**Light microscopy of *Giardia lamblia *isolated discs (A)**. Molybdate staining preparations of an isolated disc (B and C) and ultrathin sections of fractions (D and E) were visualized by TEM. We did not observe any cytoskeletal structure connected to the discs. Bar = 1 μm.

In reproducing the protocols for the isolation of the *Giardia *cytoskeleton [16], we experienced difficulties, ranging from the proper amount of detergent exposure to the best method for sample disruption; additionally, we still found flagella linked to the disc. To identify ventral disc-specific proteins, we adapted this protocol to obtain a purified ventral disc fraction. In the modified protocol, cells were resuspended in PHEM buffer to better preserve the cytoskeleton, and the detergent exposure time was reduced to 10 min. The type and time of cell disruption were also changed; instead of vortexing, we used sonication in periods of 10-15 s. With this protocol, we achieved higher amounts of isolated discs compared to previous protocols [16]. We analyzed the samples by light microscopy to assess the purity of the isolated fractions (Figure 1A). In this overview of the sample we can observe the purity of the fraction, where we found broken and intact discs. Utilizing ammonium molybdate staining (Figure 1B) and ultrathin sections (Figure 1C, D), we identified a structure of isolated and intact microtubules and microribbons by TEM. Organelles such as nuclei, peripheral vesicles and endoplasmic reticulum were not observed.

### Identification of ventral disc proteins by LC-MS/MS

**Figure 2:**
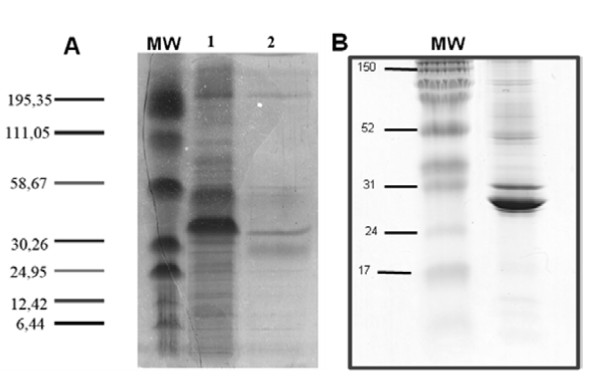
***Giardia lamblia *proteins visualized by 7,5% (A) and 12% (B) 1D electrophoresis gels**. A-1: Total proteins; A-2: ventral disc proteins. MW: molecular weight.

**Figure 3:**
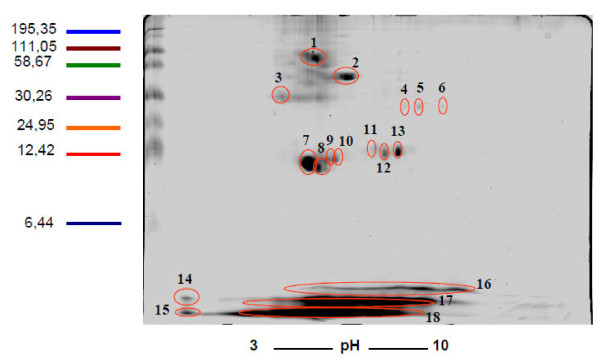
***Giardia lamblia *ventral disc proteins visualized by 2D electrophoresis gel**. The most evident spots (1 to 18) were excised and submitted to trypsin in gel digestion and LC- MS/MS mass spectrometry.

To identify *Giardia *ventral disc proteins, ventral disc fractions were separated in one-dimensional SDS-PAGE gels (7.5% and 12%) and in 2D gels (Figures 2 and 3). In a 7.5% SDS-PAGE gel, the protein profile of a *G. lamblia *ventral disc fraction showed four predominant bands ranging from 25 to 58 KDa in size. In a 12% gel, 5 predominant bands were present; however, the entire lane was investigated. In this analysis, we found known *G. lamblia *cytoskeletal proteins with molecular weights characteristic of tubulins (approximately 40 KDa) and giardins (approximately 30 KDa). The same sample was submitted to a 2D electrophoresis. Approximately 18 spots were found predominately in the neutral pH region (Figure 3). We found a group of seven proteins with molecular weights ranging from 30 to 58 KDa. Another group of eight proteins with molecular weights below 24 KDa was observed. After trypsin digestion, the four predominant bands found in the one-dimensional gel and the 18 spots from bi-dimensional gel were submitted for analysis. The results of these analyses are shown in Table 1. A total of seven proteins were identified from the 1D gel; three of these proteins, alpha- and beta-tubulin, beta- and gamma-giardin, median-body protein and SALP-1 have been previously identified as *Giardia *cytoskeletal components [17]. Several hypothetical proteins were also found, which may be targets for identification in future studies. From the 2D gel, only four spots corresponded to annotated proteins in the *Giardia *genome, the median-body protein, SALP-1, and delta- and gamma-giardins.

**Table 1:** Proteins indentified in ventral disk proteomic of *Giardia lamblia*

GEL^1^	*SPOT*	NCBI^2^	IDENTIFICATION	*SCORE*^3^	PM (KDA)^4^	PEPTIDES SEQUENCE^5^
1D	1/3/4/5/6	XP_001707372.1	Beta tubulin [Giardia lamblia ATCC 50803]	267	50701	R.INVYFNEAAGGR.Y
						R.FPGQLNADLRK.L
						K.LAVNLIPFPR.L
						R.VGEQFTAMFR.R
						R.LHFFLVGFAPLTSR.G
						K.GHYTEGAELVDAVLDVVRK.R
						R.AILVDLEPGTMDSVR.A
						K.MAATFIGNSTCIQELFKR.V

1D	1/2/3/4	XP_001706843	Alpha-tubulin [Giardia lamblia ATCC 50803]	624	49617	R.FDGALNVDLTEFQTNLVPYPR.I
						R.AVFVDLEPTVVDEVR.A
						R.QLYHPEQLISGKEDAANNYAR.G
						R.NLDIERPTYTNLNR.L
						R.LIAQCISSITASLR.F
						K.TIGGGDDAFNTFFSETGAGK.H
						R.GHYTIGKEIVDLVLDR.V
						K.EIVDLVLDR.V
						R.IHFPLCSYAPIISSEK.A
						K.LSVAELTNSVFEPANMMVK.C
						R.LIAQCISSITASLR.F
						R.FDGALNVDLTEFQTNLVPYPR.I
						R.IHFPLCSYAPIISSEK.A
						K.LSVAELTNSVFEPANMMVK.C
						K.VGINYQPPTVIPGGDLAK.V

1D	3/10	XP_001706274.1	Gamma giardin [Giardia lamblia ATCC 50803]	543	35667	K.MFQDHMVNDFRPK.F
2D	13					R.HGLQTEINSLEAIIER.E
						R.LNQEVSNFKESFDASER.N
						K.SSFSTVSSYSDHK.F
						K.DAPTTIRDSLR.L
						R.LTNAVEDPGYMTLSLISENKVDDLMFNSK.M
						R.ELESTDLRSEVAK.Q
						R.IFDAIMTTK.V
						K.AFDAENADFVGK.T

1D	3	XP_001707409.1	Median body protein [Giardia lamblia ATCC 50803] ^6^	182	100579	K.IDEYTLFMSR.S
2D	1					R.ITELEQQQR.T
						K.VNNYDQLADDKAR.L
						R.GNTSGVMAEIENIQR.Q

1D	4	XP_001705181.1	Hypothetical protein GL50803_16267 [Giardia lamblia ATCC 50803] ^6^	50	63191	K.LGELDDEALAIIR.A

1D	5/10/11	XP_001705425.1	Beta-giardin [Giardia lamblia ATCC 50803]	582	31043	R.TLTQTMDKPDDLTR.S
						K.SADNMYLTIKEEIDTMAANFR.K
						K.SLAEMGDTLNNVETNLQNQIAIHNDAIAALR.K
						K.EALKSLNDLETGIATENAER.K
						K.SLNDLETGIATENAER.K
						K.MYDQLNEKVAEGFAR.I
						R.ISAAIEKETIAR.E
						R.AVSAATTEALTNTKLVEK.C
						R.AIQEEIDREK.A

1D	5	XP_001704148.1	Hypothetical protein GL50803_13584 [Giardia lamblia ATCC 50803]^6^	61	43838	R.ATLAMELSNALDR.I R.IAELESILEGR.A

1D	11	XP_001708306.1	SALP-1 [Giardia lamblia ATCC 50803]	122	29866	R.YQHLYDAVNEQVLRR.Q
2D	8					R.AIEEERAEFTENAGMLTR.E
						R.ELETVMKYLER.N
						R.AYHSVIASYKDEIK.S
						R.ETIVAGFSDLNR.A

2D	9	ABV91519.1	Mp1p-like protein 10 [*Penicillium marneffei*]^6^	79	15571	-.VIEALLQFTNDIEKQSFQVLHK.D
						K.DVVVILQR.I
						R.IAVESQLDSR.D
						R.IAVESQLDSRDVYSLEEGFK.A
						R.DVYSLEEGFK.A

2D	9	XP_001032825.1	Hypothetical protein TTHERM_00486130 [*Tetrahymena thermophila *SB210]^6^	63	18838	K.DAGRQISILVLGK.M
						R.QISILVLGK.M
						R.QISILVLGKMPEEFFDIGK.K

2D	10	AAK32143.1	giardin delta chain [Giardia intestinalis]	147	30969	R.VTDFHEDFKR.Q
						K.IAQEHDDLLESIR.Y
						K.TSAEESFGAFIGNLTNER.N
						R.ADREQSIDEYLR.D
						K.VLAGVVAELIATR.K

Table with isolated ventral disc proteins of *Giardia lamblia*. The peptides identified were hydrolyzed with trypsin and submitted to Q-TOF and MALDI-TOF-TOF, like described in methodology

1- Gel utilized in mass spectrometry

2- National Center for Biotechnology Information: number of identification bank

3- Ideal value above 35 to efficient identification

4- Molecular weight (MASCOT)

5- Peptides to analysis based

6- These proteins were only identified in this paper and differ from other studies

## Discussion

The protein composition of the *Giardia *cytoskeleton has already been described [15,16,18]. However, these studies analyzed the whole cytoskeleton, not just the ventral disc. In their one-dimensional gel analysis, Holberton and Ward (1981) [16] found only two major bands, which correspond to proteins with approximate molecular weights of 52.5 and 30 KDa. In this work, the authors affirmed that the protein with molecular weight in the range of 50 KDa could be tubulin, as they compared the sample with the profile of purified tubulin from pig brain. Crossley and Holberton (1983) [18] isolated the cytoskeleton of *G. lamblia *and identified 20 bands from a one-dimensional gel; among these the isoforms α and β tubulin were already known. In this work, the authors named the 30 KDa protein giardin. Palm et al. (2005) [15], reproducing the protocols from Holberton and Ward (1981) [16], identified 12 spots by bi-dimensional gel. Later, proteomic analysis of this gel by mass spectrometry identified the family of giardin proteins (Alpha-1, Beta, Gamma and Delta) and two isoforms of tubulin (alpha-2 tubulin and beta-tubulin). Beyond these, a new protein, SALP-1, homologous to proteins that participate in the aggregation of striated fibers was also identified [15]. Despite the identification of the major protein families of *Giardia*, the protocol used for isolation of the whole cytoskeleton can hide the presence of less prevalent proteins that are present only in the ventral disc. Thus, to characterize and obtain greater coverage of identified proteins present only in the ventral disc we analyzed the protein profile from isolated ventral discs obtained from one- and two-dimensional gels. In the present work, with the analysis of isolated ventral disc, we identified several proteins that differ from other studies such as the median body protein. Mass spectrometry analysis identified several proteins previously known to be part of the ventral disc such as tubulin and giardins. However, the protein alpha-giardin was not identified. The absence of this protein in our analysis could be explained by the lack of relevant hits in some spots of the gel, making the proteins difficult to identify. Another consideration is that alpha-giardin might have been absent due to low amounts of this protein in the sample preparation; additionally, the isolation process could have lead to its dissociation from the structure. From the 2D gel, we also identified two proteins located in the same spot. These proteins corresponded to Mp1p, a protein found in the fungus *Penicillium marneffei*, and TTHERM, a hypothetical protein found in the ciliate *Tetrahymena thermophila*. Although these proteins could at a first moment be considered contaminants it is worth emphasizing that these proteins have described functions that could be straightly related to the disc function and structure. According to Cao et al. (1998) [19], the Mp1p protein is found in fungal cell walls and has many specific sites. Among these sites, one presents a sequence usually involved in cell adhesion processes. It is important to point out that the ventral disc is a structure that plays a vital role in the adhesion of *Giardia *to intestinal epithelial cells and inert substrates. According to Eisen and colleagues, the hypothetical protein TTHERM is homologous to tubulin [20]. In this publication of the *T. thermophila *genome, the authors believe that the genes that code for this protein are associated with the formation of the microtubule system of *T. thermophila*. Therefore, although the two proteins found are not annotated in the *Giardia *genome, they appear to be involved in biological processes related to ventral disc functions. Thus, further analysis of these proteins is necessary.

The work done by Holberton and Ward (1981) [16] was the first to isolate the cytoskeleton of *Giardia lamblia*. To observe the changes in the ventral disc during the encystation process, Palm et al. (2005) [15] adapted the procedure used by Holberton and Ward to extract the ventral disc. Here, we adapted these protocols for the isolation of the ventral disc and were able to identify the main structures of the disc, such as the microtubules and microribbons. These results were similar to those found by Holberton and Ward (1981) [16]. Additionally, we decided to work further on the isolation of the ventral disc.

The advantage of the subcellular fractionation strategy, which is based on sequential and/or density-gradient centrifugation, is that this approach allows for the separation of different structures based on their size, density and charge. In combination with mass spectrometry analysis, fractionation strategies have been extensively used to identify the molecular composition of multiple organelles and structures [21]. This strategy has been considered an effective approach to understand cellular processes and integrated cell function by decreasing sample complexity in comparison to whole cell proteomic analysis.

Proteomic analysis has been used successfully in protozoa such as *Entamoeba histolytica *[22] and *Trypanosoma cruzi *[23]. These works produced valuable information about protein localization, confirming the presence of several proteins and detecting new resident proteins. Here, we performed the proteomic analysis of the ventral disc, which is related to *Giardia *adhesion in the intestine and consequently the maintenance of infection. Identification of ventral disc proteins would enable studying ventral disc formation and changes in its structure during encystment. Further, it might identify candidates for chemotherapy as well. Also, these results can explain the dynamics of the ventral disc during adhesion to the epithelium enabling identification of proteins directly related to this process.

## Conclusions

Compared to previously published protocols, the isolation of the ventral disc shown in this work proved to be more efficient. There were smaller amounts of flagella and other cytoskeleton structures attached to the isolated discs, which improved the identification and analysis of ventral disc proteins. Proteomic analysis showed the presence of different proteins in the ventral disc which may be involved in function played by this structure in the adhesion of *Giardia *to intestinal epithelial cells, a fundamental step for the protozoan to exert its pathogenic effect and to the establishment of giardiasis.

## Methods

### Microorganisms

The *G. lamblia *WB strain was cultivated in TYI-S-33 medium [24] supplemented with 10% inactivated adult bovine serum, at 37°C for 48 to 72 h.

### Fractionation of the ventral disc

The protocol to obtain the ventral disc fraction was adapted from Holberton et al. (1981) [16]. Briefly, trophozoites were cultivated for 48 h (10^7 ^cells). After being placed on ice for 15 min, the cultures were centrifuged (Sorvall RC-5B) at 12,000 g for 20 min. Subsequently, cells were resuspended in 0.1 M PHEM buffer (60 mM Pipes, 20 mM Hepes, 10 mM EGTA and 2 mM MgCl_2_), pH 7.2, supplemented with 1% Triton X-100, at 37°C for 10 min. Parasites were disrupted by sonication on ice; an ultrasonic apparatus (Sigma, GEX 600 Model) with a standard probe for periods of 10-15 s with amplitude of 8% was used. Samples were then centrifuged at 10,000 g for 20 min, the pellets were washed with PBS buffer (0.02 M Na_2_HPO_4 _and 0.9% NaCl) and used in all the analysis described below.

### Light microscopy

Isolated ventral disc fractions were mounted between a glass slide and a coverslip and observed using a Zeiss Axio Observer D1 microscope.

### Transmission electron microscopy

For ammonium molybdate staining, sample preparations of isolated discs were placed on copper grids previously coated with 5% FORMVAR in chloroform for 2 min. The materials were then washed in 0.1 M PHEM buffer, pH 7.2 and stained with 1% ammonium molybdate for 3 min prior to observation with a Zeiss EM900 electron microscope.

For routine transmission electron microscopy preparations fractions were fixed in a solution containing 2.5% glutaraldehyde, 4% freshly prepared formaldehyde, 4% sucrose in 0.1 M PHEM buffer for 1 h at room temperature, and post-fixed in 0.1 M cacodylate buffer with 1% OsO4, 0.8% potassium ferrocyanide and 5 mM calcium chloride for 40 min. Samples were dehydrated in an acetone series and embedded in Epon. Ultrathin sections were stained with uranyl acetate and lead citrate and observed using a Zeiss EM900 transmission electron microscope.

### 1D SDS-PAGE

A total of 30 μg of ventral disc proteins were separated by both 7.5% and 12% SDS-PAGE. Each lane was arbitrarily divided into approximately 2-5 mm slices that were then subjected to in-gel proteolysis with trypsin.

### 2D SDS-PAGE

To remove salts and detergents from the samples, the samples were precipitated using a 2-D Clean-Up Kit (GE, USA). The first dimension used 18 cm strips with an immobilized pH gradient from 3 to 10. The analyzed proteins (100 μg) were dissolved in 300 μL of rehydration buffer (8 M urea, 2 M thiourea, 4% CHAPS, 40 mM Tris, 1% IPG, 0.002% bromophenol blue, and 60 mM dithiothreitol). Isoeletric protein focusing was performed using an IPGPhor instrument (GE-Health Care) with the following voltages: 30 V for 12 h, 200 V for 1 h, 500 V for 1 h, 1000 V for 1 h, 3500 V in gradient step for 30 min and 35000/18000 Vh. Between the first and second dimension, the focused strips were equilibrated for 15 min in a solution containing 50 mM Tris-HCl pH 8.8; 6 M urea; 30% glycerol; 2% SDS; 0.002% blue bromophenol (equilibrium buffer) and 6 mM dithiothreitol. Next, the strips were submitted to a second equilibrium step with an alkylation solution (2.5% w/v of iodoacetamide in equilibrium buffer) for 30 min. After equilibrium, strips were directly applied onto 12% polyacrylamide gels and electrophoresis was performed as described by the manufacturer. A Dalt six GE-Healh Care apparatus was used for 18 cm strips, and electrophoresis was conducted at 25 W per gel. Gels were then fixed and stained with Comassie Blue G 250 (Sigma-Aldrich).

### In gel digestion

1D gel sections or 2D gels spot were excised and cut into smaller pieces (approximately 2 mm). Gel pieces were destained with 25 mM ammonium bicarbonate in 50% acetonitrile for 12 h. The slices obtained from the non-reduced gels were previously reduced in a 10 mM dithiothreitol, 25 mM NH_4_HCO_3 _solution for 1 h at 56°C and then alkylated in a 55 mM iodoacetamide, 25 mM NH_4_HCO_3 _solution for 45 min in the dark. The solution was removed, and the spots were washed with 25 mM NH_4_HCO_3 _in 50% acetonitrile and then dehydrated with 100% acetonitrile. Finally, the gel spots were air-dried and rehydrated with a solution of NH_4_HCO_3 _containing 100 ng of trypsin. Gel spots were digested overnight at 37°C. The tryptic peptides were then collected in 10 μL of a solution containing 0.1% TFA and 50% acetonitrile.

### Nano LC-MS/MS mass spectrometry

The extracted peptides from the 1D SDS-PAGE gels spots were loaded into a Waters Nano Acquity system (Waters, Milford, MA). The peptides were desalted on-line using a Waters Opti-Pak C18 trap column. The sample injection volume was typically 10 μL, and the LC was performed using a nanoEase C18 150 mm × 75 μm column (Waters, Milford, MA) eluting (0.4 μL/min) with a linear gradient (10-50%) of acetonitrile containing 0.1% formic acid. Electrospray tandem mass spectra were recorded using a Q-Tof quadrupole/orthogonal acceleration Time-of-Flight spectrometer (Waters, Milford, MA) interfaced to the Nano Acquity system capillary chromatograph. The ESI voltage was set at 3500 V using a metal needle, the source temperature was 100°C and the cone voltage was 100 V. Instrument control and data acquisition were conducted by the MassLynx data system (Version 4.1, Waters); experiments were performed by scanning from a mass-to-charge ratio (*m*/*z*) of 200-2000 using a scan time of 1 s, applied during the whole chromatographic process. The mass spectra corresponding to each signal from the total ion current (TIC) chromatogram were averaged, allowing for an accurate molecular mass measurement. The exact mass was determined automatically using the Q-Tof's LockSpray™ (Waters, Milford, MA) and the Q-Tof Ultima API (Waters, Milford, MA). Data-dependent MS/MS acquisitions were performed on precursors with charge states of 2, 3 or 4 over a range of 50-2000 *m*/*z *and under a 2 *m*/*z *window. A maximum of three ions were selected for MS/MS from a single MS survey. Adduct masses of Na^+ ^and K^+ ^were automatically excluded. Collision-induced dissociation (CID) MS/MS spectra were obtained using argon as the collision gas at a pressure of 13 psi; the collision voltage varied between 18 and 45 V depending on the mass of the precursor. The scan rate was 1 scan/s. All data were processed using the ProteinLynx Global server (version 2.0, Waters). The processing automatically lock mass corrected the *m*/*z *scale of both the MS and MS/MS data utilizing the lock spray reference ion. The MS/MS data were also charge-state deconvoluted and deisotoped with a maximum entropy algorithm (MaxEnt 3, Waters).

### MALDI-TOF/TOF mass spectrometry

Roughly 0.3 μL of the extracted peptide solutions were mixed with an equal volume of a saturated matrix solution (α-cyano-4-hydroxycinnamic acid (Aldrich, Milwaukee, WI) 10 mg/mL in 50% acetonitrile, 0.1% trifluoroacetic acid) on the target plate and allowed to dry at room temperature. Raw data for protein identification were obtained on the 4700 Proteomics Analyzer (Applied Biosystems, Foster City, CA).

### Data analysis

Proteins were identified by correlation of tandem mass spectra and the NCBInr database of proteins (Version 050623, 2,564,994 sequences) using the MASCOT software (Matrix Science, version 2.1). One missed cleavage per peptide was allowed and an initial mass tolerance of 0.05 Da was used in all searches. Cysteines were assumed to be carbamidomethylated, and a variable modification of methionine (oxidation) was allowed. Identification was considered positive when at least two peptides matched with a mass accuracy of less than 0.2 Da.

## Competing interests

The authors declare that they have no competing interests.

## Authors' contributions

DL carried out the fractioning of the ventral disc, the 1D-SDS PAGE gels and the processing and analysis of mass spectrometry results, and also drafted and writes the manuscript. ISA participated in the fractioning of the ventral disc and in the processing and analysis of mass spectrometry results, produced light microscopy images and helped to draft the manuscript. LLT carried out the 2D- SDS PAGE gel, participated in the fractioning of the ventral disc and produced transmission electron microscopy images. PRG participated in the processing and analysis of mass spectrometry results. RBZ participate in the design of the study and revised it critically for important intellectual content. WS conceived of the study, and participated in its design and coordination and helped to draft the manuscript. All authors read and approved the final manuscript.
